# Epigenetic DNA Methylation Profiling with MSRE: A Quantitative NGS Approach Using a Parkinson's Disease Test Case

**DOI:** 10.3389/fgene.2016.00191

**Published:** 2016-11-02

**Authors:** Adam G. Marsh, Matthew T. Cottrell, Morton F. Goldman

**Affiliations:** ^1^Center for Bioinformatics and Computational Biology, Delaware Biotechnology Institute, University of DelawareNewark, DE, USA; ^2^Genome Profiling LLC, Helen F. Graham Cancer Center and Research Institute, Center for Translational Cancer ResearchNewark, DE USA; ^3^Marine Biosciences, School of Marine Science and Policy, University of DelawareLewes, DE, USA

**Keywords:** epigenetics, DNA methylation, Parkinson's Disease, diagnostics, blood, lymphocytes

## Abstract

Epigenetics is a rapidly developing field focused on deciphering chemical fingerprints that accumulate on human genomes over time. As the nascent idea of precision medicine expands to encompass epigenetic signatures of diagnostic and prognostic relevance, there is a need for methodologies that provide high-throughput DNA methylation profiling measurements. Here we report a novel quantification methodology for computationally reconstructing site-specific CpG methylation status from next generation sequencing (NGS) data using methyl-sensitive restriction endonucleases (MSRE). An integrated pipeline efficiently incorporates raw NGS metrics into a statistical discrimination platform to identify functional linkages between shifts in epigenetic DNA methylation and disease phenotypes in samples being analyzed. In this pilot proof-of-concept study we quantify and compare DNA methylation in blood serum of individuals with Parkinson's Disease relative to matched healthy blood profiles. Even with a small study of only six samples, a high degree of statistical discrimination was achieved based on CpG methylation profiles between groups, with 1008 statistically different CpG sites (*p* < 0.0025, after false discovery rate correction). A methylation load calculation was used to assess higher order impacts of methylation shifts on genes and pathways and most notably identified FGF3, FGF8, HTT, KMTA5, MIR8073, and YWHAG as differentially methylated genes with high relevance to Parkinson's Disease and neurodegeneration (based on PubMed literature citations). Of these, KMTA5 is a histone methyl-transferase gene and HTT is Huntington Disease Protein or Huntingtin, for which there are well established neurodegenerative impacts. The future need for precision diagnostics now requires more tools for exploring epigenetic processes that may be linked to cellular dysfunction and subsequent disease progression.

## 1. Introduction

Non-infectious diseases were once considered to be the result of faulty genes. The push of the last 50 years to decode the human genome carried the hope that we would be able to identify gene mutation errors and ultimately develop corrective gene therapies. Identifying the associations between diseases and genetic variants has been a major challenge to improving human wellness (Weitzel et al., 2016). The hope is that sequencing genomes of thousands of people and matching genome sequences to medical histories will reveal connections between genome sequence variations and root causes of diseases (Sudmant et al., 2015).

Epigenetic modifications of genomes are now recognized as playing central interacting roles in genetic determinants of health and disease (Ladd-Acosta and Fallin, 2016). Knowledge of epigenetics is essential to predict integrated genetic processes (Biémont, 2010). DNA methylation occurs on cytosines in a cytosine-guanine dinucleotide motif (CpG) and is one of the important forms of DNA epigenetic modification (Feng et al., 2010; Jones et al., 2010; Cyr et al., 2013; Nielsen et al., 2016). In general, when methylation is present gene activation is suppressed. This gene expression regulation directed by epigenetics in a large part explains why an individual is not simply a pre-programed reflection of a parentally inherited genome, but instead during their life they can develop alternative paths toward different outcomes of health and wellness.

However, there is increasing uncertainty about how to best profile site-specific CpG methylation. Despite the historical prominence of bisulfite oxidation mutagenesis as a technique, an issue of the journal *Methods* in 2015 presented multiple approaches and ideas to make these measurements (Meissner, 2015). There is a clear need and opportunity for methods development in the field epigenetics.

In this paper we present a pilot feasibility study for the development of a high-resolution DNA methylation profiling technology based on computational reconstruction of the probabilities of cytosine methylation states from next generation sequencing (NGS) data. A small trial study of Parkinson's Disease (PD) subjects was selected for application of the methylation profiling and analysis tool set. Parkinson's Disease affects more than 200,000 individuals yearly in the US. Although PD is currently incurable, medications can control symptoms. The neuropathology of symptomatic-onset PD is well established (Houlden and Singleton, 2012; Poulopoulos et al., 2012). Unfortunately, at the point where a diagnosis can be made with high confidence, neurodegeneration has generally progressed 5–10 years (Miller and O'Callaghan, 2015). Thus, most intervention therapies post-diagnosis have limited efficacy to alter PD progression because heavy neuronal damage has already caused up to 60–80% loss of nigral dopamine neurons (Zwagerman and Richardson, 2013; Miller and O'Callaghan, 2015). There is an urgent need to develop early diagnostic PD biomarkers that would enable clinicians: (i) to intervene at the beginning of the course of neurodegeneration, and (ii) to monitor progress of therapeutic treatment responses in individual patients.

Given recent insights into potential methylation biomarkers in PD (Masliah et al., 2013; Pihlstrøm et al., 2015; Tan et al., 2016), we apply a novel NGS approach to profile blood DNA that reveals subtle but quantitative shifts in epigenetic DNA methylation profiles correlated with early symptomatic diagnoses. Blood was selected as the genomic DNA source because of the ease of collection that would make a screening or diagnostic test easy and accessible to all patients. We know lymphocytes are the largest source of DNA in whole blood (10^6^ cells per ml) and recent work has shown that circulating lymphocytes in PD patients evidence quantifiable changes in cellular activities (Masliah et al., 2013; Alberio et al., 2014; Colasanti et al., 2014; Grozdanov et al., 2014; Ide et al., 2015; Tan et al., 2016). Thus, we apply a novel high-resolution, quantitative methylation measurement technology to test the efficacy of this approach to reveal epigenetic profiles of PD in blood.

## 2. Materials and methods

### 2.1. Sample processing

A small sample size was chosen for this initial feasibility study as a 3 vs. 3 comparison of early-age PD males (symptomatic in their mid-30's) and healthy males (*n* = 6). Our logic was that if a blood-based epigenetic signal could to any degree be ascertained in a cohort of only six individuals, then it would justify the time, effort and expense of pursuing a much larger validation study. Thus, the goal of this project was to simply assess if a methylation signal could be discerned.

For this pilot study, blood serum samples from the Coriell Institute Biorepository (www.coriell.org) were selected for three males with early-age Parkinson's Disease (#ND20108, #ND24665, and #ND28170). Three healthy males in the same age and demographic groups were selected as controls (#ND16294, #ND19290, and #ND24642). Descriptions of each patient sample are available via the Coriell Institute's online catalog system. Genomic DNA (1 μg each individual; comprised mostly of B-cells) was shipped directly to commercial sequencing providers. Sequence data reported here were obtained from the Yale Core Center for Genomic Analysis (Illumina HiSeq 2500; New Haven, CT).

Fragmentation of the gDNA samples followed standard Illumina protocols except for an additional restriction enzyme digest step at the beginning of the work flow. A 1 μg aliquot of the DNA was first cut with a single methyl-sensitive restriction endonuclease, HpaII. Once digested, DNA was washed with Qiagen's QIAquick PCR Purification Kit and sheared to a median size of 300 bp using a Covaris AFA sonicator. DNA libraries were prepared using Illumina's Sample Prep Kits, and 71-cycle single-read sequencing was performed on genomic DNA libraries using Illumina's sequencing by synthesis (SBS) technology. Fragmentation, library preparation and quality control checks were all performed by the sequencing provider using industry standard protocols.

### 2.2. Methylation quantification

Overall, our strategy was derived from an earlier approach for assessing DNA methylation in the late 1990's that used differential restriction fragment profiles to assess cytosine methylation sites (Reyna-López et al., 1997). Over the last decade, we have developed a computational approach to harness the power of methyl-sensitive restriction enzymes with the base-pair specific precision of NGS to statistically reconstruct CpG site-specific methylation in heterogenous cell population samples (Marsh and Pasqualone, 2014). In the hg19 reference genome, there are 2.4 million HpaII sites that can be quantified simultaneously in this approach. In general, this approach falls into the category of “MSRE-Seq” methods. There have been other methodological implementations of methylation-sensitive assays like this, most recently “DREAM” in 2012 (Jelinek et al., 2012; van Esterik et al., 2015; Bouwmeester et al., 2016), but the challenge of assessing the probability of methylation events from NGS sequence data across whole-genomes has limited earlier work from being successfully adopted in the field beyond a few laboratories.

Quantification of CpG methylation profiles and subsequent statistical processing were performed on a commercial bioinformatic pipeline and software platform (Genome Profiling LLC, Newark, DE). The gDNA preparation protocol is non-mutagenic and when coupled with the analytics platform enables concurrent gene variant and epigenetic analyses from a single NGS run. Both GATK (Broad Institute, Cambridge, MA) and Issac (Illumina, San Diego, CA) variant call pipelines have been used to extract SNP and INDEL data from these NGS fastq files in addition to the quantitative methylation profiles. Ultimately these high-resolution measurements of fractional methylation at CpG sites across a genome provide a deep data resource for integrating profile data with potential functional shifts in specific genes from know pathways.

#### 2.2.1. Software platform

The integrated platform workflow is managed via a shell pipeline running python and R scripts, starting from raw-read QC filtering of fastq sequence files to final analyses of methylation profiling differences between patient groups. The platform performs the following tasks: (1) quality control to filter sequence tags for >97% read confidence, (2) isolation of target sequence reads, (3) sequence compression to reduce complexity, (4) read alignment to hg19 reference genome, (5) CpG quantification for 5'-methyl-cytosine site distributions, (6) methylation profiling comparison between patient groups, and (7) output of data plots, tables and analyses in a report package. The platform consists of a series of python scripts optimized for distributed processing on a multi-core server. All analyses were performed using virtual machines under Amazon's Web Services cloud infrastructure.

There are two metrics recovered from the raw sequence read files that are based on independent characteristics of DNA fragmentation via restriction digests and random shearing and local scale differences in coverage bias. One metric is proportional to the probability that a CpG site is fully methylated in a heterogenous sample (all gDNA copies present in the sample extraction) and the other is related to the probability that the same CpG site is fully unmethylated. These metrics are derived for each CpG site and are utilized in subsequent statistical analyses. This multi-measurement approach provides greater statistical sensitivity and robust error control. Using ordination analytics to partition measured differences in CpG states into discrete vectors (e.g., non-metric multidimensional scaling; see below) allows for the identification of the most significant shifts in CpG methylation states among subject groups.

All sequence data reported here are publicly available by contacting the lead author or through the Sequence Read Archive of the US National Center for Biotechnology Information (Accession #PRJNA342035).

### 2.3. Analyses

#### 2.3.1. Methylation load

Analytics are executed at three levels of comparison: individual CpG_[*i*]_ methylation, individual genes by summation across measured CpG sites, and higher-order pathways by assessing methylation status of component genes. This is the concept of methylation load across a defined sequence (Ordway et al., 2007; Hogenbirk et al., 2013) and serves to highlight functional differences that may exist when comparing different subject groups. Here, we utilize Differential Methylation Load (Δ*ML*), which is a summation of site-specific differences in CpG methylation that are then summed across a gene or gene domain or combination of genes (pathways). This is simply calculated as the signed sum of the difference in %MET scores for each CpG site within the defined gene or region being scored:

(1) $$\Delta ML_{j}=\sum_{i}\;\;CpG_{norm[i]}-CpG_{PD[i]}\;\;$$

where the differential methylation load of the *jth* gene or pathway is calculated across all *ith* CpG sites contained within that gene or pathway. This difference is ‘signed’ and always calculated as *NORM*−*PD* so that positive numbers reflect higher methylation loads in healthy males and negative numbers reflect higher methylation in the PD subjects.

#### 2.3.2. Differential methylation

One approach employed here utilizes differential gene expression (DGE) analyses using the R package, *edgeR*, (Bioconductor) (Robinson et al., 2010; Zhou et al., 2014). Using DGE tools for methylation analysis is common with other open source tools, like *RnBEADS* (Assenov et al., 2014), where thousands of quantitative variables are compiled across a much smaller number of samples (< 50). Methylation score data is well suited for such an analysis because of the limited range of response (two orders of magnitude) in the quantitative variables in contrast to the higher range of responses that exist in DGE data sets. Also, these DGE analytics packages have very well developed false-discovery rate calculations and adjustment procedures. Briefly, a methylation data table containing normalized “counts” (i.e., methylated cytosine counts normalized to total cytosines at specific CpG sites) for all six sample libraries was loaded into an edgeR library in R. These data were used as the source from which a DGEList (Digital Gene Expression List) object was created. Differential response between two sample groups was calculated for single CpG sites using a site-specific false discovery rate correction applied to each pairwise comparison.

#### 2.3.3. Ordinate analyses

An ordination procedure called non-metric multidimensional scaling (NMDS, Legendre and Legendre, 2012) analysis was used to analyze integrated pattern discrimination in methylation profiles. This technique is like a principal components analysis. The primary difference between DGE and NMDS is that DGE analysis is executed at a pairwise level across separate, individual CpG methylation score values, while in NMDS all CpG sites for each sample are integrated as one pattern and the patterns are then compared across samples to identify CpG sites that are conserved within a sample group while also divergent between different sample groups. The R package *vegan* contains several robust ordinate analyses.

#### 2.3.4. Hierarchical clustering

To assess pattern similarities between individual CpG sites, we used the R package *pv-clust* to run a hierarchical clustering tree with iterative bootstrap support (*n* = 1000) using only the top 40 CpG sites that provided the most discriminating power between sample groups in the NMDS analysis.

#### 2.3.5. Graphics

The R package *ggplot2* is extremely versatile at handling a broad range of data types and graphic formats (Wickham, 2009). The Perl module *circos* is an essential tool for representing multiple data tracks at very dense genomic scales (Krzywinski et al., 2009).

## 3. Results and discussion

### 3.1. Methylation quantification and profiling analyses

Interest in DNA methylation profiling technologies has grown rapidly in recent years. One of the first methods reviews appeared only 6 years ago (Laird, 2010). Within the last year, one issue of the journal *Methods* was devoted entirely to DNA methylation profiling methods, highlighting the variety of work that is under development (Meissner, 2015). The *Methods* review pointed to several gaps in current approaches, the key being the transition from qualitative to quantitative data. In addition, comparing results between studies can be problematic when different profiling methods are used. Epigenetic profiling research is wide open for development of standard clinical diagnostic methods. The broad application of a standard approach would advance the quantitative understanding of the role of epigenetics in many diseases.

To be effective, screening tests for disease risks should utilize non-invasive sampling of easily obtained tissues. In addition, genetic and epigenetic profiles would be obtained and interpreted together, yielding a comprehensive genomic assessment. Recent work has shown the relevance of integrating DNA methylation profiles and DNA sequence analysis obtained from blood (both cell-free and cell fractions) (McClay et al., 2015). Here we assess the efficacy of a computational platform based on a methyl-sensitive restriction enzyme approach to recover epigenetic information in blood relevant to a known disease state in early-age Parkinson's Disease.

It is important that a methylation profiling approach also yields variant calls (SNPs) with the same efficiency as a commercial standard whole genome sequence (WGS) profile. We used the “Genome in a Bottle” (GIAB) gDNA standard that has been developed by the US National Institute of Standards (NIST) to be used as a reference for assessing genome variant sequence analysis pipelines (Cornish and Guda, 2015). There were only minor differences in the number of SNPs recovered from a standard WGS library preparation performed by Macrogen USA and our restriction digest library protocol (Figure S1). Both methods resulted in identifying 94% of the known SNP sites in GIAB across all chromosomes. Of those SNP sites, both methods yielded greater than 99% concordance with the known GIAB genotype. Thus, the fragmentation step via restriction digests during library synthesis does not impact the downstream efficiency of variant call analyses.

The repeatability of replicate measures of CpG methylation measurements between different replicate libraries is important. Two samples (two replicates each) were assayed on independent sequencing lanes on a HiSeq X10 (Macrogen USA). Measurement precision was expressed as the coefficient of variation (CV), which represents a percentage deviation from the mean (*n* = 2 independent libraries) for each CpG site scored by our analytical platform (Figure S2). The frequency distribution of precision for duplicate gDNA samples reveals that over 70% of CpG sites have a CV deviation that is less than 2.5%. A cumulative frequency distribution shows that overall 9~0% of the CpG sites were scored with a CV that is less than 10%, indicating that the quantitative methylation metrics are highly repeatable.

**Figure 1:**
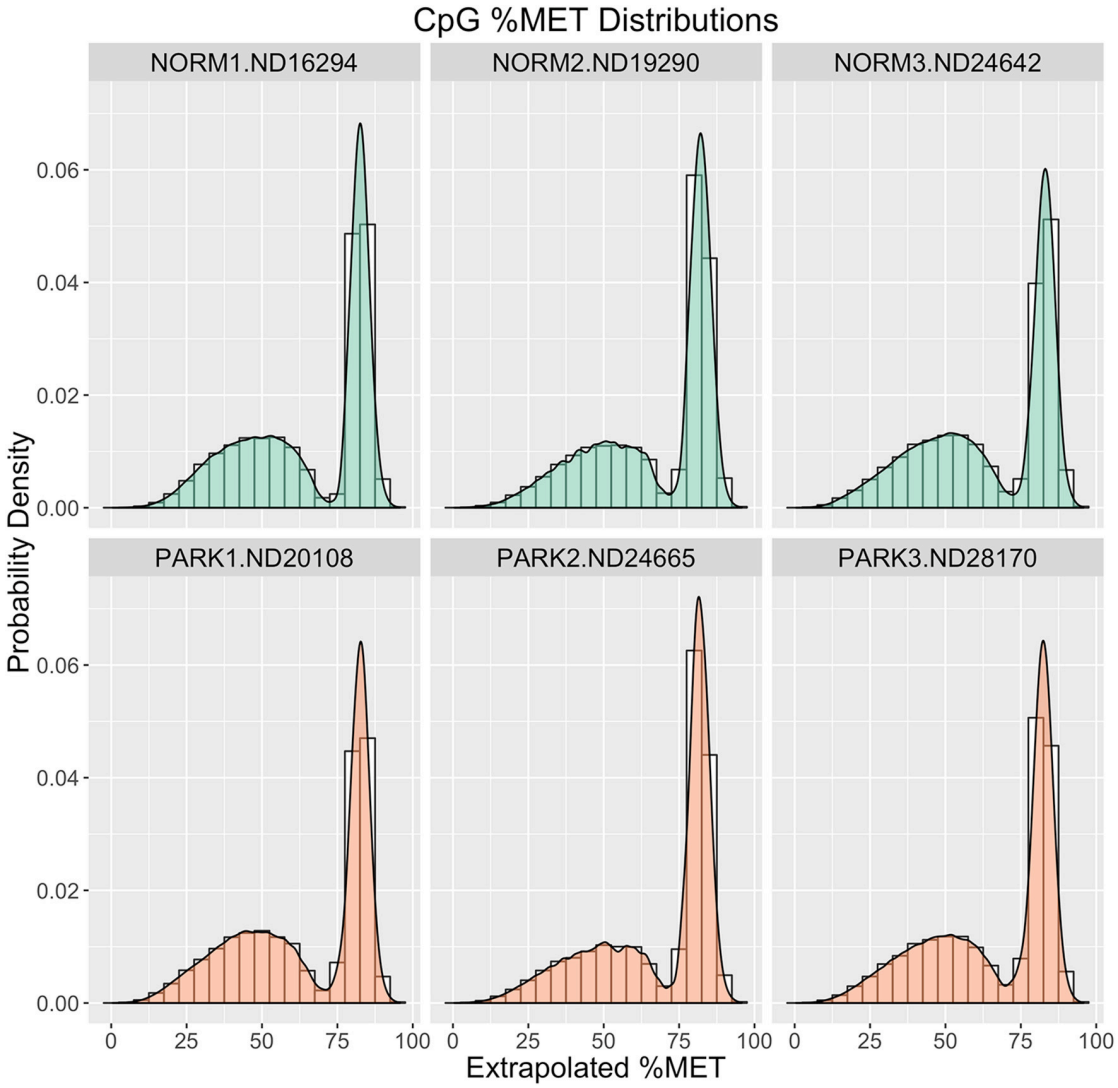
**Methylation (%) Frequency Distributions**. Sample profiles for %MET as a frequency distribution (histogram) overlaid with a probability density distribution function (filled logistic curve). Orange = Parkinson's Patients. Green = Normal Healthy Males.

To assess raw CpG measurements, Figure 1 compares frequency distributions of methylation scores for each individual. Here we see that the blood gDNA samples are distinctly bimodal for all individuals. There is a sharp peak of CpG sites centered around 90% methylation. This peak comprises about one third of the CpG observations for each sample. A second broader peak is evident around 60% methylation and comprises the remaining two thirds of the distribution in each sample. At this broad level of comparison there is no apparent difference between these healthy normal and Parkinson's Disease males.

The human reference genome (hg19) has 13 million CpG sites, including 2.4 million CpGs found in the sequence CCGG, which is the recognition sequence for HpaII. For analyses, a quick filter for each CpG site was executed to identify those for which the group means differed by more than 10%. The final data matrix consisted of 504,373 CpG sites. Because most of the HpaII CCGG sites are located in or near defined gene bodies, this data set yielded a deep level of gene-specific profiles.

As a first pass to assess potential patterns in methylation between groups, the CpG data matrix was merged with functional annotation information for each individual CpG site, combining gene information bed files with KEGG functional classification nomenclature (Kyoto Encyclopedia of Genes and Genomes). Using this integrative information, we explored potential functional impacts of these CpG patterns that could be related to disease stress or symptoms. This *post hoc* processing is best described as data mining because it leads to formulation of hypotheses about early disease phenotypes, rather than specific conclusions about possible mechanisms. This is true especially in light of the small number of individuals in this exploratory pilot study with the primary goal of assessing efficacy of this CpG methylation profiling platform.

**Figure 2:**
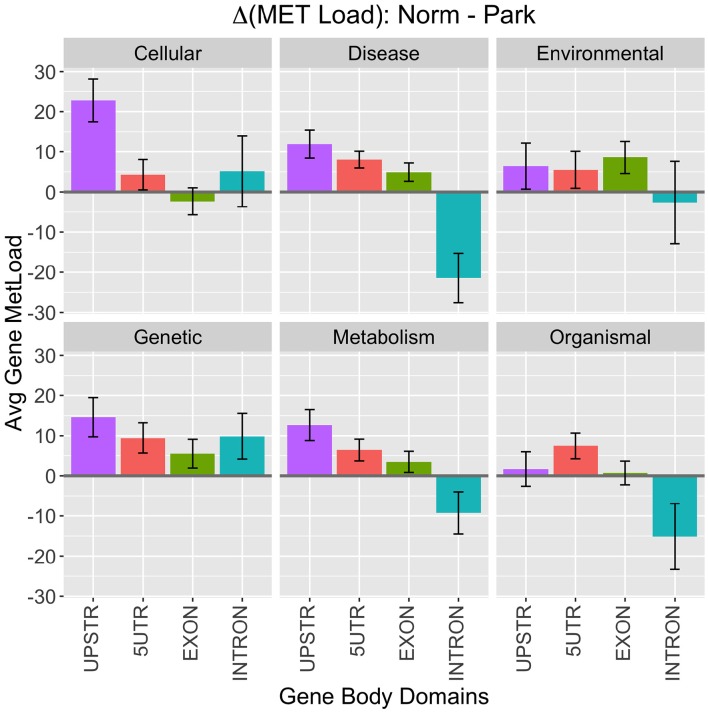
**KEGG Class Processes**. High-level breakdown of methylation load (Δ*ML*) among functional KEGG class designations and gene body domains. Positive numbers indicate higher methylation in healthy males and negative numbers indicate higher methylation in Parkinson's Disease patients. Values plotted are means ± SE across the number of genes in each category.

An agglomerative metric was used to assess differential CpG methylation status between PD and healthy subjects at higher genomic levels. Differential methylation load (Δ*ML*) was calculated as a summation of the difference in %MET scores for each CpG site within a defined region or gene group being scored (Equation 1). Broad levels of methylation were assessed for KEGG functional classes and gene body domains (Figure 2). Here, methylation load was distinctly different across gene domains and across functional KEGG groups. A large difference appears in Cellular Processes where 3 kb-upstream promoter domains of genes were more hypomethylated in PD individuals, suggesting functional differences in gene expression regulation within this broad gene/pathway group. Another noticeable difference appears in the Disease Response class where introns in this gene group are more hypermethylated in PD individuals. This class contains genes involved with many cell surface recognition and cell signaling processes predominant in lymphocytes. Overall, methylation loading on genes between PD and healthy individuals was not equivalent across domains and functional KEGG groups. Thus, there are distinct differences to be be pursued at lower levels of analysis.

### 3.2. Individual CpG methylation patterns

Distinct differences in Figure 2 warrant a detailed CpG site-specific analysis. The R package *edgeR* was utilized because of the strong false discovery rate correction (FDR) that is calculated for each single CpG site comparison. Only minor adjustments in the input matrix were needed to translate the methylation scores into a format appropriate for a DGE analysis. The resulting differential library sizes for the six samples ranged from 1.71 to 1.77 M with a common dispersion factor of 0.034. Using an FDR adjustment (Benjamini and Hochberg, 1995), rather than a fixed threshold, the site specific dispersion estimates ranged from 0.001 to 0.231 with a median value of 0.035.

An exact pairwise *T*-test (*edgeR*) was performed using dispersion adjusted CpG variances (FDR threshold 0.05). Results for the top 40 CpG metrics sorted by *P*-value are shown in Table 1. In total, 1008 CpG sites were statistically significant at the *p* < 0.0025 level (after FDR adjustment). Group differences were evenly split with 499 sites showing higher methylation in PD males while 509 sites had higher methylation in healthy males. Table 1 shows how the top distinguished sites compare in terms of log fold change ratios around 5.0 and highly significant FDR *P*-values. It is important to point out that the analysis employed the two metrics that are generated for each CpG site from the analytics platform instead of a single extrapolated % methylation value. These paired metrics are designated by a prefix “M” or “U” in the table and indicate values that are proportional to the probability that a CpG site is uniformly methylated on all genome copies in the sample and the probability that the site is uniformly unmethylated among all genome copies, respectively. Overall, these results suggest there is a small, defined set of CpG sites whose methylation status discriminates *post hoc* between healthy and PD males. The fact that these differences were detected in blood is concordant with other studies (Masliah et al., 2013; Alberio et al., 2014; Colasanti et al., 2014; Grozdanov et al., 2014; Ide et al., 2015; Tan et al., 2016).

**Table 1:** **Top 40 CpG Sites by *P*-value**.

**CpG Site**	**logMET**	**logFC**	**std *P*-val**	**FDR *P*-val**	**Response**
M.chr19.0050595864	1.569	5.122	1.091e-20	4.438e-16	up
M.chr1.0148864870	1.305	5.131	1.641e-15	3.338e-11	up
M.chr8.0007469535	−1.209	5.122	1.384e-13	1.877e-09	down
M.chr5.0002151560	1.214	5.103	2.238e-13	2.276e-09	up
M.chr6.0161066486	1.275	4.997	4.233e-13	3.444e-09	up
M.chr6.0153451650	1.183	5.068	1.317e-12	8.930e-09	up
M.chr9.0045413153	−1.106	5.151	6.130e-12	3.563e-08	down
M.chr17.0079876105	1.113	5.094	1.007e-11	5.119e-08	up
M.chr8.0029207347	−1.134	5.039	1.207e-11	5.455e-08	down
M.chr13.0028499045	1.120	5.068	1.388e-11	5.646e-08	up
M.chr17.0027071641	−1.091	5.116	2.104e-11	7.405e-08	down
M.chr8.0011538630	1.109	5.075	2.184e-11	7.405e-08	up
M.chr7.0102330910	−1.106	5.082	2.522e-11	7.458e-08	down
M.chr3.0125648119	−1.105	5.063	2.567e-11	7.458e-08	down
M.chr10.0048186138	1.082	5.130	2.778e-11	7.533e-08	up
M.chr13.0045992725	1.101	5.058	3.484e-11	8.482e-08	up
M.chr2.0180871406	−1.116	5.035	3.545e-11	8.482e-08	down
M.chr2.0109834011	1.076	5.108	3.841e-11	8.680e-08	up
M.chr19.0033167362	1.106	5.046	4.095e-11	8.768e-08	up
M.chr4.0013536107	−1.095	5.075	4.624e-11	8.957e-08	down
M.chr13.0057715641	1.084	5.077	4.624e-11	8.957e-08	up
M.chr13.0021872770	−1.093	5.058	5.437e-11	1.005e-07	down
U.chr19.0050595864	−2.129	4.644	6.241e-11	1.059e-07	down
M.chr12.0130662189	1.081	5.069	6.249e-11	1.059e-07	up
M.chr9.0138980742	1.070	5.065	8.435e-11	1.372e-07	up
M.chr2.0152954469	1.075	5.034	1.018e-10	1.592e-07	up
M.chr3.0062364171	1.051	5.090	1.261e-10	1.901e-07	up
M.chr8.0049468828	−1.084	4.993	1.658e-10	2.409e-07	down
M.chr1.0234950494	1.058	5.052	2.059e-10	2.873e-07	up
M.chr10.0030723853	−1.065	5.035	2.122e-10	2.873e-07	down
M.chr20.0040246428	1.040	5.102	2.189e-10	2.873e-07	up
M.chr20.0037357841	−1.061	5.022	2.499e-10	3.177e-07	down
M.chr3.0147129204	1.039	5.071	2.672e-10	3.294e-07	up
M.chr4.0134072763	1.058	5.008	2.943e-10	3.521e-07	up
M.chr20.0062588163	1.034	5.069	3.578e-10	4.158e-07	up
M.chr5.0072861609	−1.030	5.072	4.060e-10	4.588e-07	down
M.chr7.0072494504	−1.006	5.150	4.569e-10	4.925e-07	down
M.chr17.0000029728	−1.023	5.085	4.601e-10	4.925e-07	down
M.chr7.0099149631	1.016	5.111	4.991e-10	5.206e-07	up
M.chr10.0090751531	1.038	5.022	5.146e-10	5.234e-07	up

*Rank ordered listing of CpG sites by P-values that have been adjusted for the maximum false discovery rate (FDR) threshold using the Benjamini-Hochberg algorithm in the R package “edgeR.” Site-specific dispersion was estimated to adjust CpG variances instead of using an overall sample FDR adjustment. Analysis utilizes independent methylation metric scores and not extrapolated %methylation values. The “Response” colummn indicates: **up** (= higher) and **down** (= lower) methylation in normal healthy males relative to the Parkinson's patients. logMET = log[2] of the primary methylation metric scores*;

*logFC = log[2] of fold change*.

**Figure 3:**
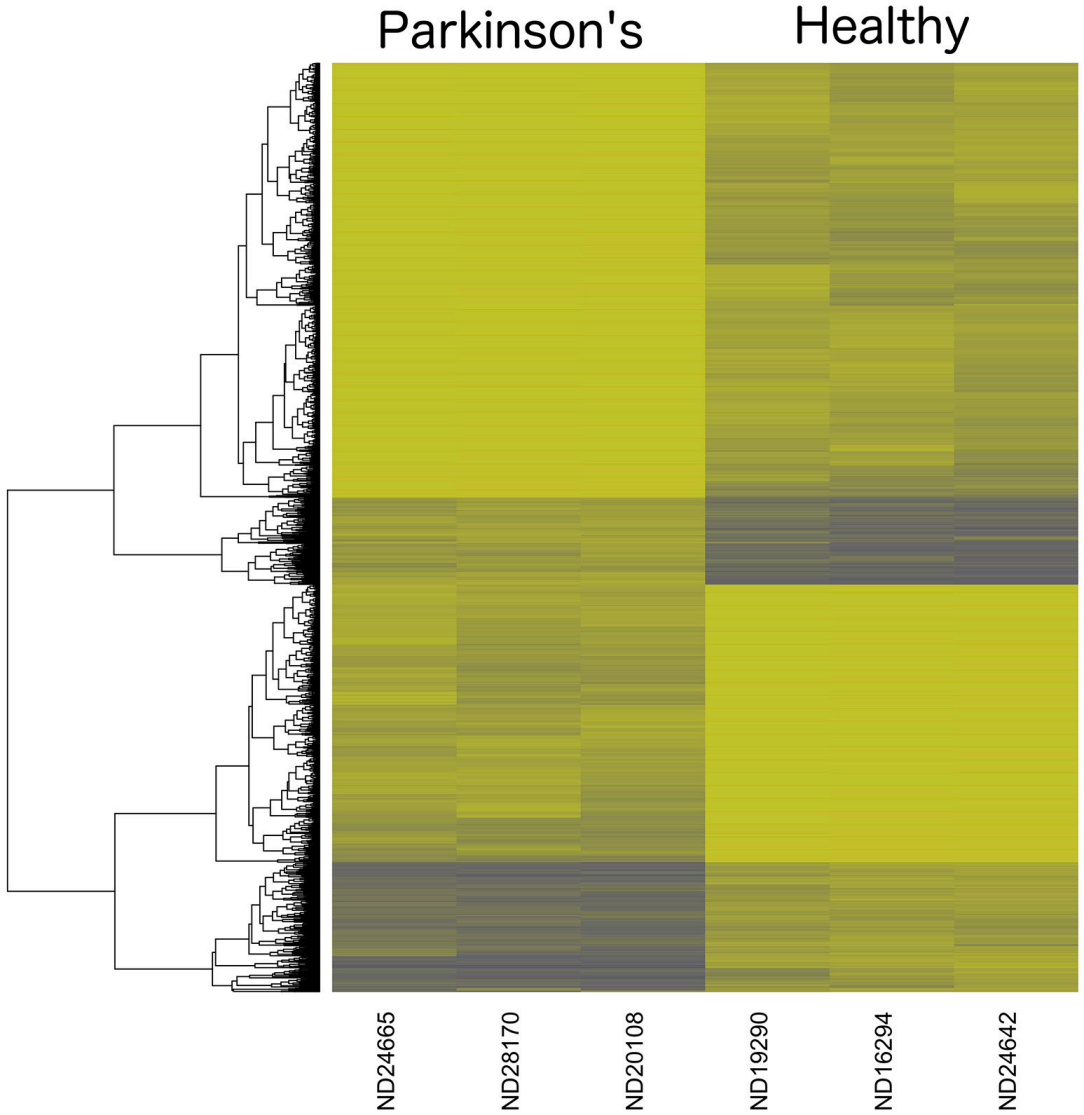
**Dendrogram Heatmap of CpGs: *p* < 0.0025**. Focusing on the most statistically significant CpG site changes, there are 1008 CpG sites with a methylation score difference with a probability of a type-I error of *p* < 0.0025 (after applying a false-discovery rate adjustment). The resolution between Parkinson's Disease patients and healthy males reveals quantitative separation among these groups of CpG sites. Each row represents the score values for a single CpG_[*i*]_ site, with a hierarchical clustering association based on % methylation across all six individual samples in the study.

A heatmap of CpG methylation score patterns was generated with the R package *gplots* using the *heatmap.2* function. This routine provides clustering across both rows (CpG_[*i*]_) and columns (samples) with cell values represented on a green to blue color scale (Figure 3). Only CpG sites with an FDR adjusted *P*-value < 0.0025 were used. Stark visual differences in color blocks point out two main contrasts: (1) low signal to mid-signal, and (2) mid-signal to high signal. These contrasts arise in comparing both PD to Healthy and Healthy to PD, so there were four response groups of CpG sites evident in total. The color differentiation in Figure 2 underscores the low *P*-values in Table 1. Essentially, low sample variances in CpG measurements within a group (PD vs. healthy) establish a statistical foundation upon which error rates are extremely low.

A challenge with high-dimensional data sets arises from concerns about applying pairwise comparisons across thousands of non-independent data observations. To avoid this, we pursued a second analysis approach based on group pattern recognition, rather than multiple individual observation comparisons. Using the same input matrix as in the DGE based analysis, we applied an ordinate analysis technique, similar to principle component analysis, that employed an iterative algorithm solution using similarity rankings of CpG sites and repetitively applied coefficients to the derived response variables to locate a sample CpG data vector in a mutlidimensional space (Legendre and Legendre, 2012).

**Figure 4:**
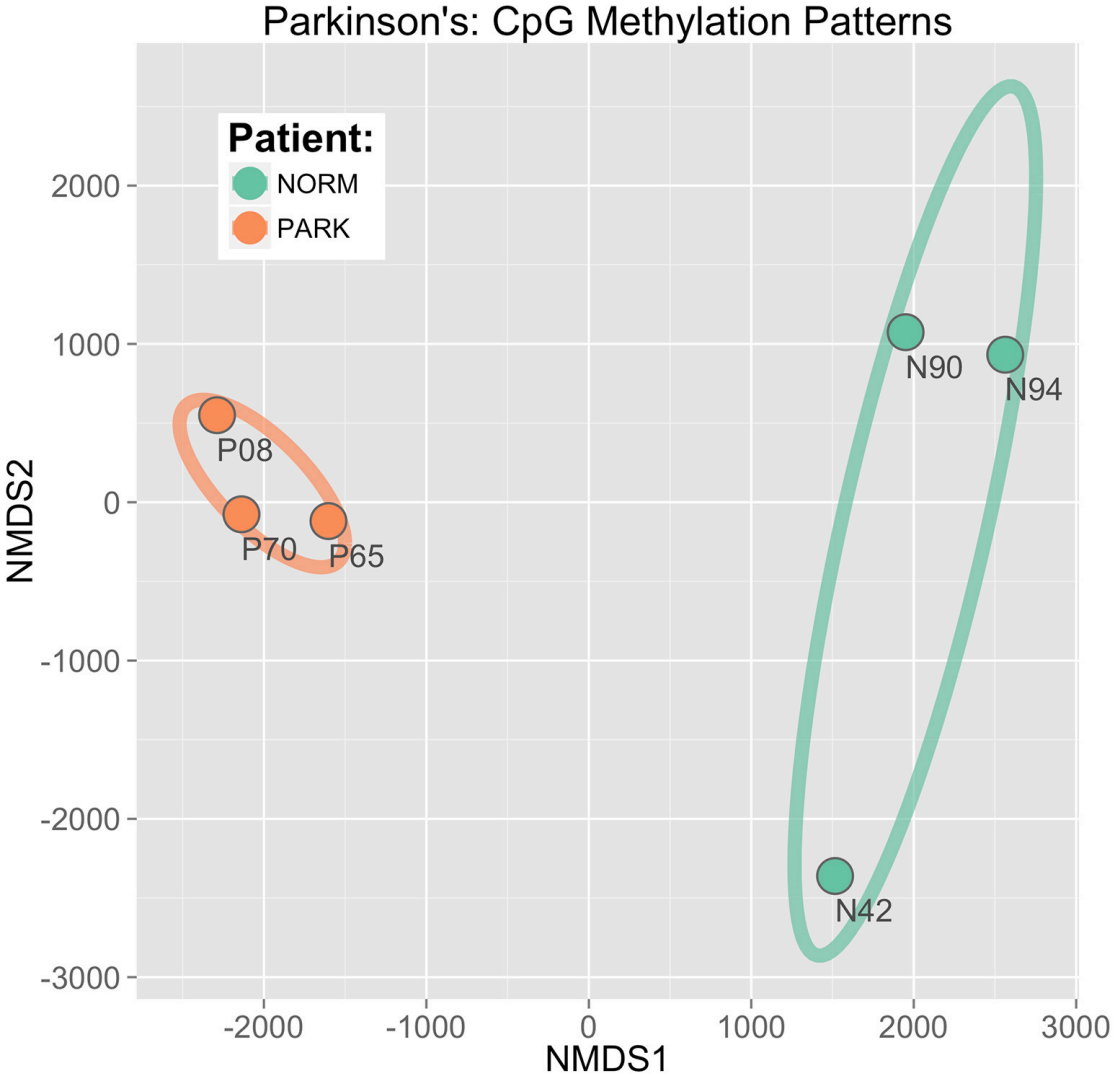
**CpG discrimination of Parkinson's individuals from healthy individuals**. Using an ordinate analysis technique of non-metric multidimensional scaling (NMDS), CpG methylation profiles were compared among individuals to isolate patterns conserved within groups while also differing between groups (Parkinson's patients vs. normal healthy males). The first two component axes are plotted to locate the individual sample points in a 2D plane. Ellipses drawn represent 95% Confidence Intervals around the location of the true group means.

This approach, called non-metric multidimensional scaling (NMDS), established a clear and direct discrimination of methylation score profiles of PD and healthy males. The two sample groups are distinctly separated with the first two ordinate dimensions plotted as *x, y* coordinates (Figure 4). Here, differential CpG methylation patterns discriminated between healthy vs. PD epigenetic profiles with high confidence. The ellipses in the figure represent 95% confidence intervals around the true group mean locations in the ordinate space. The spatial gap between the ellipses indicates that there is a very low probability that this separation between groups could have arisen by chance alone. Iterative bootstrap analysis of randomized data reveal a statistical probability of *p* < 0.0001. This separation shows a distinct, conserved DNA methylation shift evident in the blood profiles between PD and healthy males. The tight co-localization of the PD sample points in the NMDS plane indicates a strong and consistent cytosine methylation profile that is shared among all three PD patients, while the profiles of the heathy individuals reveal higher interindividual variation.

**Figure 5:**
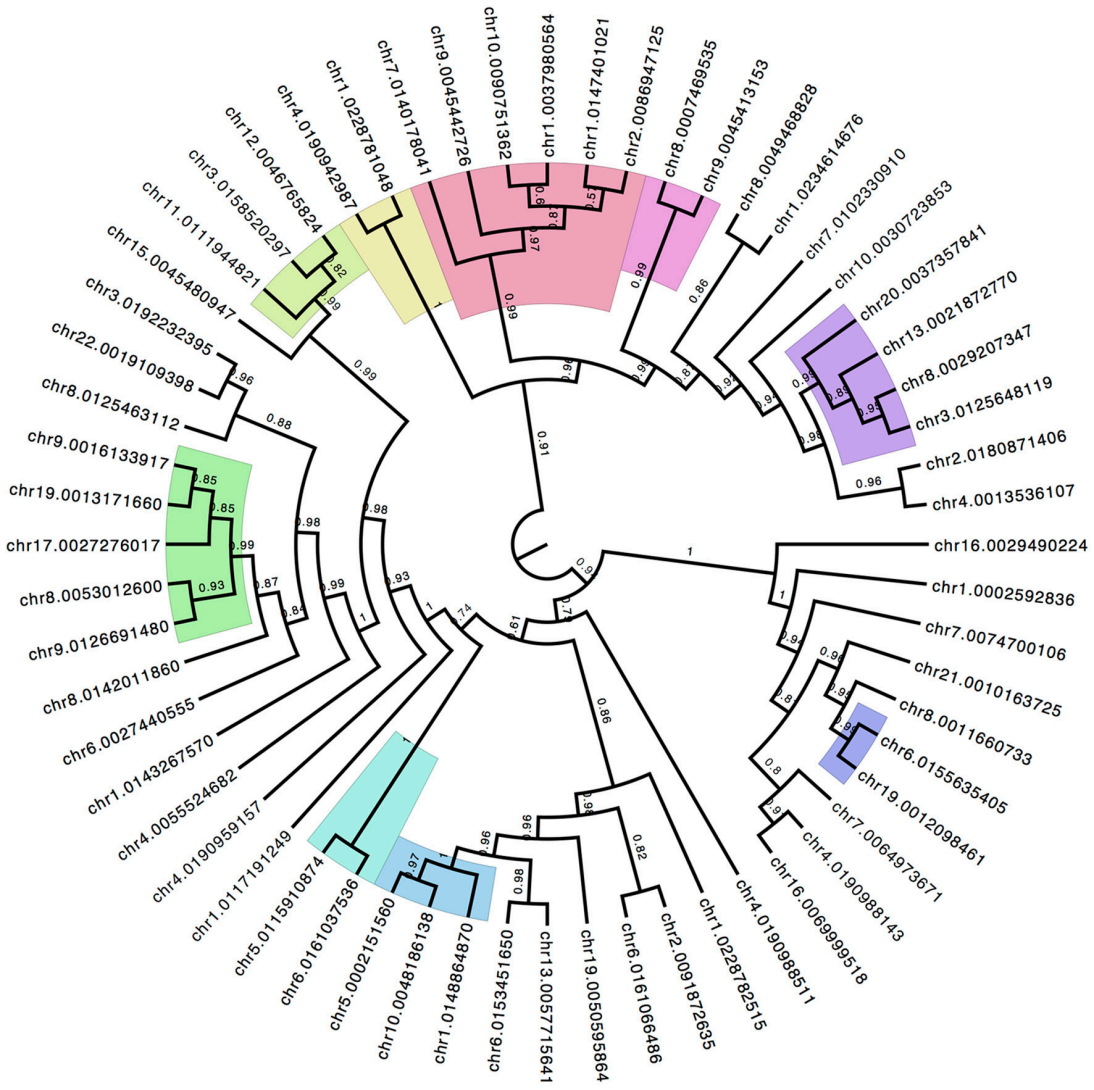
**Hierarchical Clustering of Top Discriminating CpG Sites**. The top ranked CpG sites that contribute to the group discrimination in the NMDS ordinate analysis are isolated and compared for similarity in methylation patterns across all individuals. Iterative boot-strap analysis (1000 reps) using the R package *pv-clust* established confidence levels of support for branches. Color shading represent the lowest order branch with significant association supported by >97% of the runs.

The NMDS analysis does not produce a *P*-value for significance of individual CpG sites because it is not a multiple-pairwise test for significance. It is a combined pattern analysis technique to establish the overall discrimination of the underlying data matrix to separate between patient groups. However, the contribution of each individual CpG site to the overall separation between sample groups can be recovered and allows for a ranking of the discrimination power of individual sites. Using the top 60 ranked CpG sites a hierarchical clustering with bootstrap replication was performed (Figure 5) to identify parallel patterns of methylation shifts across samples. The color shades in Figure 5 highlight lowest branches at which replicate support is >97%. There are several distinct groupings of CpG sites. The clustering indicates that the pattern of methylation among these groups is highly similar and may suggest a hypothesis of coordinate mechanisms of methylation status.

### 3.3. Gene level methylation patterns

Clustering top discriminating CpG sites based on methylation patterns (Figure 5) may indicate potential sites with similar mechanisms of regulating methylation/demethylation. To assess whether such cluster associations indicate parallel methylation will require an analysis of how methylation shifts between subject groups compare at higher-levels of cell functioning. First, a methylation load summation (Δ*ML*) was utilized to compare methylation levels across defined genes between patient groups. A list of the top 40 hypo-methylated genes in PD males (Table 2) provides a look at genes that are the most under methylated relative to healthy males. Because of the general release of transcriptional gene controls associated with hypo-methylation, we can postulate that these genes are likely more active in PD than in healthy controls, but direct testing is necessary before further inferences can be made.

A top 40 list (Table 2) is just a beginning point for an analysis with sufficient patient sample size (*n* = 20 to 50) to warrant digging deeper to look for markers and mechanisms of disease phenotype progression. The total number of annotated genes for which a Δ*ML* score could be calculated was 4044. In a concerted biomarker study, an investigator would be able to parse deeper into ranked gene lists to assess the functional relevance of smaller shifts in methylation load. Also important to consider in a deeper study is the break out of Δ*ML* into gene body structures, such as introns (miRNAs, transcript splice variants), CpG Islands (coordinated local domain shifts, retrotransposons), and 2 kb upstream promoters (transcriptional regulation). With a sufficient patient sample size, a break-down Δ*ML* could be evaluated separately in different functional contexts within, or in proximity to, a defined gene body. Thus, total Δ*ML* across an entire gene is a high-level integrated score that reveals “genes of interest,” and focus research attention on gene body targets for which an assessment of methylation shifts should be executed on a finer-scale.

**Table 2:** **Top 40 Hypo-methylated Genes in Parkinson's patients**.

**MethyLoad**	**UCid**	**Pfam**	**RefSeq**	**Pathway**
1293.2	uc002lwd.2	unk	MIR7850	Alcoholism
1068.1	uc002pbu.2	Rad10	MIR6088	Fanconi.anemia.pathway
928.6	uc002ydf.3	unk	NTSR1	Neuroactive.ligand-receptor.in
881.4	uc002xtw.1	RhoGEF	PREX1	Chemokine.signaling.pathway
820.6	uc002ybs.3	TAFH	TAF4	Herpes.simplex.infection
694.1	uc002hhu.3	ASC	ASIC2	Taste.transduction
605.2	uc002ikv.3	unk	WNT3	HTLV-I.infection
538.2	uc003wjq.1	CHGN	CHPF2	Glycosaminoglycan.biosynthesis
525.1	uc002kog.2	unk	RAB31	Endocytosis
520.5	uc002lov.3	unk	CDC34	Herpes.simplex.infection
519.5	uc002wnw.2	DSL	JAG1	Notch.signaling.pathway
513.2	uc002hgn.1	unk	RAB11FIP4	Endocytosis
494.4	uc002fwo.4	VWA	ITGAE	Regulation.actin.cytoskeleton
492.8	uc002wmu.1	unk	BMP2	Basal.cell.carcinoma
486.7	uc001zng.2	DSL	DLL4	Notch.signaling.pathway
469.9	uc001mil.1	Adrenomedullin	Adrenomedullin	Vascular.smooth.muscle.contrac
467.7	uc010gke.1	Neur_chan_LBD	Neur_chan_LBD	Nicotine.addiction
459.3	uc002xqz.3	Peptidase_M10	MMP9	Hepatitis.B
444.2	uc002lry.2	Ephrin	EFNA2	Axon.guidance
433.6	uc001ack.2	NtA	AGRN	ECM-receptor.interaction
428.0	uc001uii.3	Fz	FZD10	HTLV-I.infection
412.7	uc002jqm.1	unk	galanin.re	Neuroactive.ligand-receptor.in
395.7	uc002ewa.3	Exo_endo_phos	SMPD3	Sphingolipid.metabolism
369.6	uc003xhm.3	Y_phosphatase	DUSP4	MAPK.signaling.pathway
357.6	uc002mmq.1	Laminin_G_2	COL5A3	Amoebiasis
357.2	uc002sgf.3	unk	PCBP1	Spliceosome
348.5	uc003xpx.4	unk	HGSNAT	Lysosome
344.8	uc021pxg.1	FGF	FGF8	Melanoma
343.5	uc003keo.3	unk	F2RL1	African.trypanosomiasis
341.8	uc002kwg.2	unk	CDH2	Arrhythmogenic.right.ventricul
334.1	uc002yet.2	Neur_chan_LBD	LOC100130587	Nicotine.addiction
333.2	uc001uew.3	SET	KMT5A	Lysine.degradation
330.1	uc001hqd.4	WGR	PARP1	Base.excision.repair
328.0	uc002xaa.3	ESCRT-III	CHMP4B	Endocytosis
323.6	uc010asv.1	Laminin_G_1	Laminin_G_1	Cell.adhesion.molecules.(CAMs)
312.4	uc002glw.4	Laminin_EGF	NTN1	Axon.guidance
312.2	uc003yhp.3	TGF_beta	GDF6	Cytokine-cytokine.receptor.int
310.1	uc002kmv.1	Band_41	EPB41L3	Tight.junction
305.2	uc010wnn.1	Dynein_light	DYNLL2	Vasopressin-regulated.water.re
299.5	uc003kei.1	unk	synaptic.v	ECM-receptor.interaction

*Genes are rank ordered by methylation load score (ΔML) and are presented with PFAM and RefSeq name designations (where available) as well as KEGG pathway associations. The gene list has been filtered for functional pathway assignments. Hypothetical genes or genes with unannotated functions were not included in this rank listing*.

Overall, there is a noticeable representation of genes involved with cell surface signaling events, cell-surface interactions, and even neuron functioning. Keeping in mind that the primary source of gDNA in these samples are B- and T-cells, plus much smaller amounts of circulating cell-free DNA, the strong “immune” based response suggests that in blood a traceable signal indicative of PD exists at a functional gene level and not just as independent CpG methylation events. A KEGG pathway descriptor is included in the table but many genes are active in several pathways and thus the primary descriptor does not always relate to the most proximal function a gene may contribute to via altered methylation states in this specific study. Given the small number of individuals and the range of annotation possibilities, the presented pathways should be considered as hypotheticals.

Similarly, a list of the top 40 hyper-methylated genes in PD males (Table 3) provides a look at genes that are the most over methylated relative to healthy males, and thus likely to be less active or repressed. Again there is an intriguing list of genes associated with cell signaling, surface recognition, cellular defense and neuron function. To assist in parsing through these gene lists (Tables 2, 3) to identify significant features we employed a custom PubMed (NCBI) literature citation tool to screen all indexed publications on Parkinson's Disease and/or neurodegeneration. Instead of trying to manually assess each gene in the list, this approach uses PubMed as a knowledge base and efficiently determines literature support for each gene in both tables in association with Parkinson's Disease.

**Table 3:** **Top 40 Hyper-methylated Genes in Parkinson's patients**.

**MethyLoad**	**UCid**	**Pfam**	**RefSeq**	**Pathway**
−1707.0	uc001vqx.3	Collagen	MIR8073	Amoebiasis
−1188.1	uc004cfb.2	zf-C4	RXRA	Hepatitis.C
−802.5	uc004ays.3	7tm_3	GABBR2	Morphine.addiction
−770.9	uc011auw.2	Sec7	IQSEC1	Endocytosis
−761.9	uc003apg.3	Myosin_head	MIR6819	Viral.myocarditis
−748.4	uc001vqw.4	unk	COL4A1	Amoebiasis
−712.4	uc003bvw.3	unk	MIR378B	Pancreatic.secretion
−630.0	uc001vmw.4	HS6ST	MIR4501	Glycosaminoglycan.biosynthesis
−604.7	uc011awl.2	unk	LOC105376997	Non-small.cell.lung.cancer
−581.0	uc001qzz.3	SAM_PNT	ETV6	Transcriptional.misregulation.
−571.0	uc021xkv.1	HEAT	HTT	Huntington's.disease
−565.9	uc001urt.2	unk	PDX1	Maturity.onset.diabetes.the.yo
−446.0	uc001mjo.2	Glycos_transf_2	GALNT18	Mucin.type.O-Glycan.biosynthes
−441.4	uc003iwc.3	Phosphodiest	ENPP6	Ether.lipid.metabolism
−435.9	uc003bkv.4	Sema	PLXNB2	Axon.guidance
−407.8	uc003qvl.3	unk	FGFR1OP	Chemokine.signaling.pathway
−404.2	uc003bye.1	unk	WNT7A	HTLV-I.infection
−394.5	uc003jcm.3	Acyltransferase	LPCAT1	Ether.lipid.metabolism
−392.1	uc001lsx.1	unk	MUC2	Amoebiasis
−384.3	uc003nzl.2	Fibrinogen_C	CYP21A2	Focal.adhesion
−379.9	uc003eux.4	FATC	ATR	HTLV-I.infection
−369.6	uc003pvk.3	unk	FYN	Measles
−360.5	uc001mhu.3	unk	WEE1.homol	Cell.cycle
−349.8	uc003qmg.3	HS2ST	LOC105378047	Glycosaminoglycan.biosynthesis
−339.0	uc031scj.1	unk	LINC01014	Vascular.smooth.muscle.contrac
−333.9	uc003aps.2	unk	CACNG2	Dilated.cardiomyopathy
−323.7	uc021ywr.1	RYDR_ITPR	ITPR3	Alzheimer's.disease
−323.7	uc003pwh.4	Sulfotransfer_1	HS3ST5	Glycosaminoglycan.biosynthesis
−321.8	uc001ykv.4	HATPase_c	HSP90AA1	Prostate.cancer
−320.6	uc003bia.3	DAGK_cat	CERK	Sphingolipid.metabolism
−316.5	uc010smh.1	Tubulin_C	TUBA1C	Pathogenic.Escherichia.coli.in
−314.5	uc001tuk.1	WWE	DTX1	Notch.signaling.pathway
−312.1	uc001ojv.3	unk	RHOD	Axon.guidance
−304.5	uc001rbt.2	Lig_chan	GRIN2B	Alcoholism
−288.9	uc011kgj.1	14-3-3	YWHAG	Epstein-Barr.virus.infection
−285.6	uc010lee.1	unk	SEMA3D	Axon.guidance
−284.8	uc001luc.2	unk	CTSD	Tuberculosis
−283.9	uc003fwy.4	Pkinase_Tyr	PAK2	Renal.cell.carcinoma
−278.8	uc001oph.3	FGF	FGF3	Melanoma
−275.0	uc011bwl.1	unk	actin.bind	Axon.guidance

*Genes are rank ordered by methylation load score (ΔML) and are presented with PFAM and RefSeq name designations (where available) as well as KEGG pathway associations. The gene list has been filtered for functional pathway assignments. Hypothetical genes or genes with unannotated functions were not included in this rank listing*.

**Figure 6:**
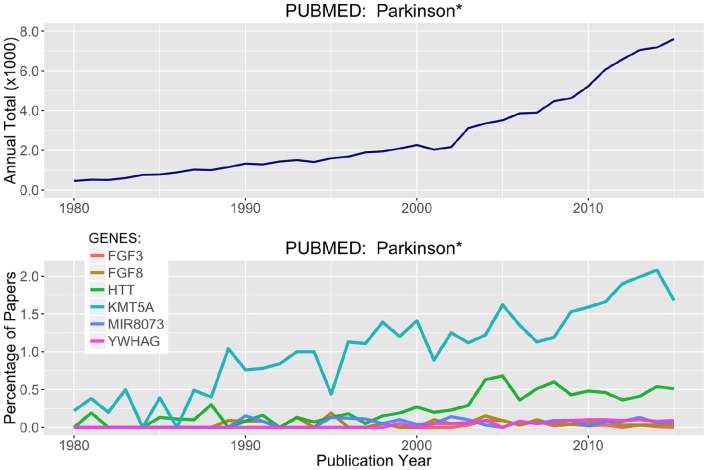
**Literature Citations for Parkinson and Neurodegeneration**. The NCBI publication database PubMed was searched for research articles using the words Parkinson^*^ or neurodegenerat^*^ between the years of 1980 and 2015. **(Top)** Total publications recorded by year matching these search criteria. **(Bottom)** Using rank ordered genes from Tables 2, 3, the top methylated genes were used to search the PubMed citations and identify which of these highly differentially methylated genes were associated with prior research on neurodegenerative diseases and processes.

Parsing through the PubMed citations (inclusive of Abstracts), a threshold of >100 total publications was used to hunt for genes in Tables 2, 3 that have been prominently linked to or associated with Parkinson's Disease or neurodegeneration or neural function. Figure 6 presents the most prominent genes identified: FGF3, FGF8, HTT, KMTA5, MIR8073, and YWHAG. These genes all show substantial differential methylation in this study as well as being prominently represented in the literature with some connection to PD and/or neurodegeneration. In Figure 6, the field of PD research has exponentially increased from 1980 to 2016. The representation of genes in this publication group are shown as percent of total publication values, so the percentage of publication numbers in the lower panel are normalized to the exponential increase already, and increases in the percentage values are indicative of real increases in the representation of specific genes within this literature.

Of the six genes in Figure 6, two stand out more prominently. First, KMT5A or SETx, is a protein N-lysine methyltransferase that monomethylates both histones and non-histone proteins. Histone 4 (H4K20) is a target and is methylated during mitosis and represents a specific tag for epigenetic transcriptional repression (Kalakonda et al., 2008; West et al., 2010; Malik et al., 2015). Second, HTT, is Huntington Disease Protein or Huntingtin, for which there are well established neurodegenerative actions (Gusella et al., 2014; Kaliszewski et al., 2015; Kumar et al., 2015; Labadorf and Myers, 2015). Simple literature based searches are not definitive and there is a great deal of language complexity when parsing through abstracts targeting in on just a few specific words. However, a knowledge based approach is one of the most efficient ways to tackle problems like this where long lists of genes (1000's) can be efficiently scored for topical relevance.

**Figure 7:**
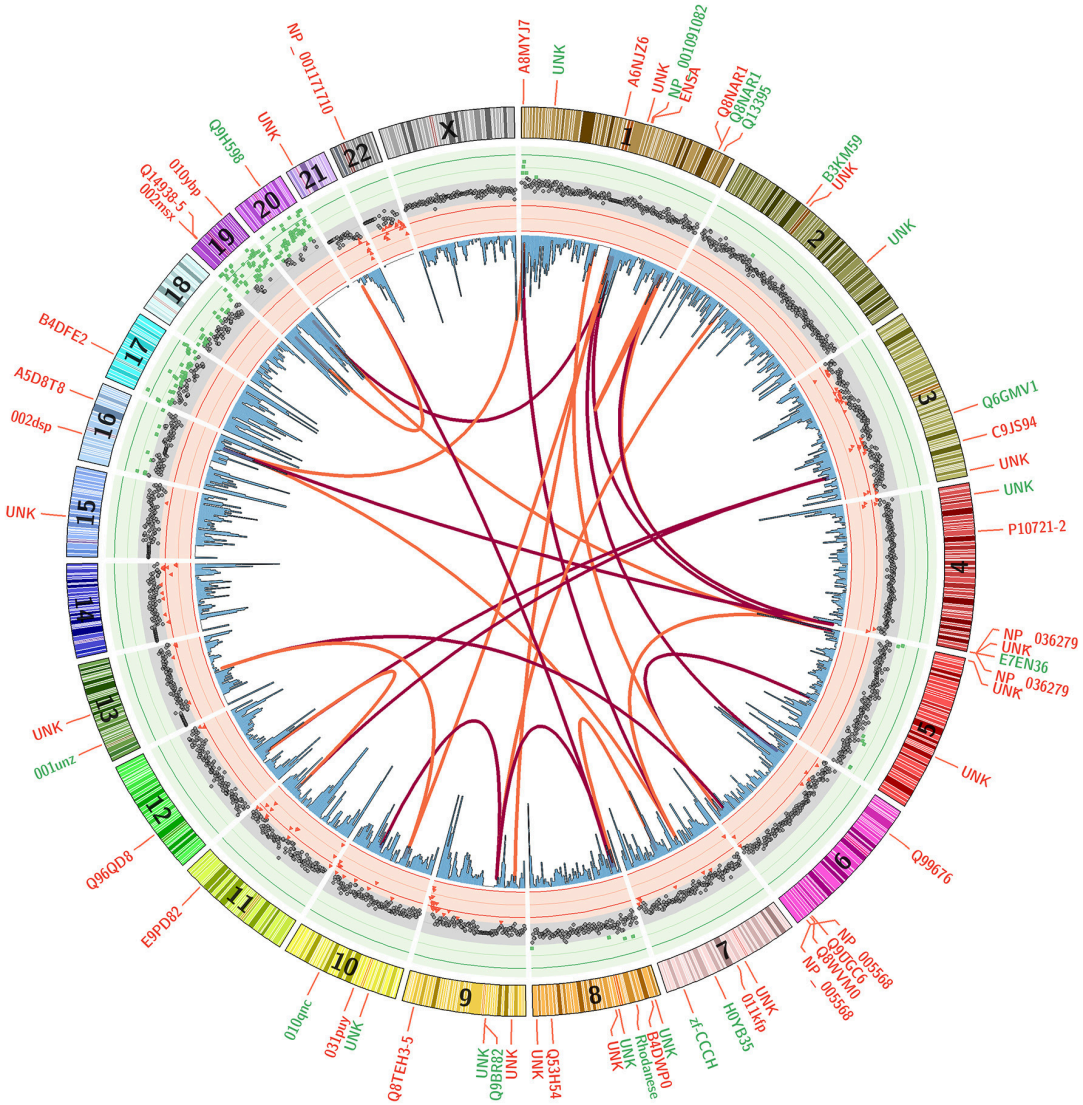
**Differential Methylation Load with Top 20 CpG Sites**. A mean subtraction of CpG methylation scores (Healthy minus Parkinson) was used to calculate a summation methylation load score across genomic domains (Δ*ML*). These data are presented as a scatterplot with red and green background to accentuate areas where they are most different. Blue histogram bars present CpG densities in gene promoter domains. The correlative association between top 20 CpG sites are shown as red arcs that track the first highest correlation for each CpG site while orange arcs show the second highest. Gene labels indicate the loci positions of the top 60 discriminating CpG sites, with red indicating higher methylation in Parkinson's patients and green indicating higher methylation in healthy males.

An ideogram figure combines differential methylation load, CpG gene promoter densities, CpG clustering associations, and impacted genes (Figure 7). Here, (Δ*ML*) is presented as the outer track scatter plot and clearly shows how the vast majority of chromosome domains (at 1 MB intervals) are equivalently methylated between both groups (gray points). There are only a very few regions where differential methylation is substantially evident (red or green points), and these hyper- and hypo-methylation deviations appear mostly in regions where CpG density is highest in promoter regions.

The arcs in Figure 7 present the clustering similarities for just the top 20 CpG sites (for visual clarity). The red arcs connect CpG sites that are the most closely correlated, the orange arcs connect second-best correlations. It is interesting to note that the highest correlated CpG sites do not occur in the same gene or chromosomal region. Their locations are separated across chromosomes, but these CpG sites are rarely located in domain regions where there is a large Δ*ML* between groups. The genes in which the top CpG sites are located are labeled around the outside of the ring, color coded by whether that CpG site is more methylated in the healthy group (green) or more methylated in the PD group (red). Overall, mapping the changes across methylation states helps to assist in identifying potential or rational hypotheses for “mechanism of action” differences between patient groups.

The differential methylation responses of CpG sites integrated into a genome profile (Figure 7) shows the overlaying of multiple data tracks that are necessary to bring some focus to genomic-based differences. All these variables come into play in piecing together the epigenome profile and assessing the distinguishing characteristics that could define PD processes/risk apart from healthy subjects. This integrated plot helps to pin-point associations not evident in the large data tables to suggest associations, correlations and response mechanisms that we can mentally explore and determine if there are observational patterns that warrant further focus.

### 3.4. PD markers

The slow progression of many neurodegenerative diseases leaves a window of time for therapeutic interventions that could potentially ameliorate disease symptoms, retard advance of the disease and/or even cure the disease. There is a clear advantage for early detection and intervention. To facilitate early diagnoses, the hunt for markers of Parkinson's Disease has traditionally focused on behavioral, cognitive and motor activity profiling (Mehta and Adler, 2016). Within the last decade, work is progressing to identify quantitative molecular signals in blood, cerebral spinal fluid, and other peripheral tissues to identify any systemic traces of neurodegenerative stress or activity in source tissues that could be readily obtained for a screening assay (Schneider et al., 2016).

The idea that a neurodegenerative disease signal can be detected within peripheral tissues other than the cerebral cortex is well established. There are active efforts looking at skin tests to detect signs of PD via alpha-synuclein (SCNA) in epidermal nerves (Donadio, 2014; Donadio et al., 2016; Rodríguez-Leyva et al., 2016). Even gastric and colonic mucosa have been profiled for markers (Chung et al., 2016). Blood is probably the most active area of PD marker research looking at a range of potential targets from IGF-I titers (Bernhard et al., 2016), micro-RNA signatures (Ding et al., 2016), mitochondrial densities (Pyle et al., 2016), vitamins (Ding et al., 2013; Ide et al., 2015), lymphocytes (Tan et al., 2014, 2016) and proteins like alpha-synuclein (SNCA) (Pihlstrøm et al., 2015).

As the field of epigenetics has rapidly developed, some attention has turned to assessing the potential for this genetic-based chemical information system to serve as a diagnostic source for PD (Ai et al., 2012). Pioneering work has been conducted by Desplats et al. (2011) describing an interaction between SCNA and DNMT1 (methylation maintenance) that likely contributes to the broader hypomethylation shifts in neurons of PD patients. Shifts in DNA methylation are a definite component of PD. The work of Pihlstrøm et al. (2015) shows correlated methylation of SNCA from both cerebral cortex and blood within PD patients, so the SCNA signature of the disease can be traced in blood. Likewise, Masliah et al. (2013) demonstrates genome-wide correlates of genome methylation patterns between both brain and blood samples in PD patients. An epigenetic signal linkage is evident between brain and blood in PD. The ability to assess epigenetic proximal signals of neurodegeneration in peripheral tissues establishes the utility of using blood as a tissue source for PD marker assays. An epigenetic response within peripheral blood cells to PD progression within patients is demonstrable, especially in circulating cells like lymphocytes (Tan et al., 2014, 2016). The functional role of white blood cells moving through circulatory and lymph systems makes them an effective sentinel target for disease response markers as they perfuse through tissues in a high state of signal sensitivity monitoring micro tissue environments.

Despite the apparent utility of assaying for DNA methylation markers in PD, there are only a handful of studies published in the literature in the last 5 years pursuing this avenue of research. The main limitations seem to be methodological problems with accurately quantifying gene methylation and the cost of NGS based assay tools needed to cover large patient cohorts. Although this present study is a feasibility test of a DNA methylation platform, the low variance and high discrimination achieved suggest that circulating lymphocytes in PD subjects may provide gene-based epigenetic markers of disease states. The majority of these markers are not likely to be proximally related to PD symptoms, but instead secondarily related to the stress and response of the body to the presence of neurodegenerative processes. This disease signal is likely to be unique in terms of an immune system response to maintain an individual's current state of health.

## 4. Conclusions

An important synthesis of Figure 7 is the potential understanding of how methylation signals may transfer molecular information from single CpG sites to genes to pathways to cellular function. This cross level linkage is an important evidentiary source for triaging a list of “most-informative” CpG sites that could be combined into a targeted panel assay. The real end goal is to utilize early-stage epigenetic shifts that may indicate changing disease risks as a clinical screening tool. The first step is to combine 96 CpG sites into a high-throughput qPCR screening platform (technologies like Fluidigm, NanoString) and begin testing for efficacy among subjects with defined disease phenotypes. We are not at that point with this study here, but we are a step closer to that point with the technology platform that has generated the data presented.

At a functional level, we can trace patterns in differential methylation back to pathways and genes that support hypothesis-level ideas about potential roles of DNA methylation in altered molecular, cellular and physiological activities of PD. These “signals” are evident in blood even though they convey a distinct neurodegenerative signature. Even with a small pilot cohort, we statistically recover epigenetic profiles that discriminate between healthy and PD blood profiles, with a functional relevance in many of the comparative differences. The quantitative sensitivity and repeatability (across patients) of the DNA methylation metrics presented here are substantial enough to warrant further exploration with larger patient cohorts and with the goal of trying to establish a blood-based marker screening test for early age Parkinson's Disease.

Overall, the results of this profiling work reveal a large number of differentially methylated CpG sites that can be identified with high statistical confidence. With a larger subject cohort, these results would support the design and development of a targeted panel assay of select CpG sites using qPCR measurement platforms. Ultimately, targeted panels for detecting shifts in site-specific CpG methylation could make it possible to diagnose early onset stages of impending diseases or disease risks by identifying pre-symptomatic genomic changes via epigenetic mechanisms.

## Author contributions

This work was conceived and designed by: AM and MG. AM performed the experiments. MC and AM analyzed the data. MG, AM and MC contributed reagents/materials/analysis tools. AM and MC wrote the first draft of the paper. All authors read and approved the final draft.

## Funding

The development of this work was supported by grants from the US National Science Foundation: #0944557 to AM, #1316055 to co-PI AM, and #1355306 to AM. Additional funding from Genome Profiling LLC covered costs of sequencing and computation.

### Conflict of interest statement

The technology and software platform was developed by AM in his capacity as a professor of Computational Biology and Bioinformatics at the University of Delaware. The IP is licensed by Genome Profiling LLC from the University. Authors AM, MC and MG declare financial interests in Genome Profiling LLC.

## Supplementary material

Figure S1 **Efficiency of Genome Variant Analyses**. The NIST standard “Genome in a Bottle” was processed using a commercial sequencing lab (Macrogen USA) comparing standard whole genome library preparation and downstream variant analysis (WGS) to a HpaII fragmentation prior to library preparation and then normal sequencing and variant analysis (GenPro). Both methods recovered 94% of the known SNP variants in the Genome in a Bottle genotype.

Figure S2 **Replicate Precision**. The gDNA from two separate samples were each split and used for replicate library preparations and NGS sequencing runs. **(A)** Frequency distribution of the observed Coefficient's of Variation (CV) for individual CpG sites. **(B)** Cumulative frequency plot showing that almost 90% of the measured CpG sites in the replicates had a CV < 10%.

## References

[B1] Ai S. Shen L. Guo J. Feng X. Tang B. (2012). DNA methylation as a biomarker for neuropsychiatric diseases. Int. J. Neurosci. 122, 165–176. 10.3109/00207454.2011.637654 22115417

[B2] Alberio T. McMahon K. Cuccurullo M. Gethings L. A. Lawless C. Zibetti M.. (2014). Verification of a Parkinson's Disease protein signature in T-lymphocytes by multiple reaction monitoring. J. Prot. Res. 13, 3554–3561. 10.1021/pr401142p 24946097

[B3] Assenov Y. Müller F. Lutsik P. Walter J. Lengauer T. Bock C. (2014). Comprehensive analysis of DNA methylation data with RnBeads. Nat. Methods 11, 1138–1140. 10.1038/nmeth.3115 25262207 PMC4216143

[B4] Benjamini Y. Hochberg Y. (1995). Controlling the false discovery rate: A practical and powerful approach to multiple testing. J. Roy. Stat. Society. Ser. B 57, 289–300.

[B5] Bernhard F. P. Heinzel S. Binder G. Weber K. Apel A. Roeben B.. (2016). Insulin-like growth factor 1 (IGF-1) in Parkinson's Disease: potential as trait-, progression- and prediction marker and confounding factors. PLoS ONE 11:e0150552. 10.1371/journal.pone.0150552 26967642 PMC4788352

[B6] Biémont C. (2010). From genotype to phenotype. what do epigenetics and epigenomics tell us? Heredity 105, 1–3. 10.1038/hdy.2010.66 20551983

[B7] Bouwmeester M. C. Ruiter S. Lommelaars T. Sippel J. Hodemaekers H. M. van den Brandhof E.-J.. (2016). Zebrafish embryos as a screen for DNA methylation modifications after compound exposure. Toxicol. Appl. Pharmacol. 291, 84–96. 10.1016/j.taap.2015.12.012 26712470

[B8] Chung S. J. Kim J. Lee H. J. Ryu H.-S. Kim K. Lee J. H.. (2016). Alpha-synuclein in gastric and colonic mucosa in Parkinson's disease: limited role as a biomarker. Move. Disord. 31, 241–249. 10.1002/mds.26473 26686342

[B9] Colasanti T. Vomero M. Alessandri C. Barbati C. Maselli A. Camperio C.. (2014). Role of alpha-synuclein in autophagy modulation of primary human T lymphocytes. Cell Death Dis. 5:e1265. 10.1038/cddis.2014.211 24874737 PMC4047919

[B10] Cornish A. Guda C. (2015). A comparison of variant calling pipelines using genome in a bottle as a reference. Biomed Res. Int. 2015:456479. 10.1155/2015/456479 26539496 PMC4619817

[B11] Cyr A. R. Hitchler M. J. Domann F. E. (2013). Regulation of SOD2 in cancer by histone modifications and CpG methylation: closing the loop between redox biology and epigenetics. Antiox. Redox Signal. 18, 1946–1955. 10.1089/ars.2012.4850 22946823 PMC3624766

[B12] Desplats P. Spencer B. Coffee E. Patel P. Michael S. Patrick C.. (2011). Alpha-synuclein sequesters Dnmt1 from the nucleus a novel mechanism for epigenetic alterations in lewy body diseases. J. Biol. Chem. 286, 9031–9037. 10.1074/jbc.C110.212589 21296890 PMC3059002

[B13] Ding H. Dhima K. Lockhart K. C. Locascio J. J. Hoesing A. N. Duong K.. (2013). Unrecognized vitamin D-3 deficiency is common in Parkinson disease Harvard Biomarker Study. Neurology 81, 1531–1537. 10.1212/WNL.0b013e3182a95818 24068787 PMC3888173

[B14] Ding H. Huang Z. Chen M. Wang C. Chen X. Chen J.. (2016). Identification of a panel of five serum miRNAs as a biomarker for Parkinson's disease. Parkinson. Relat. Disord. 22, 68–73. 10.1016/j.parkreldis.2015.11.014 26631952

[B15] Donadio V. (2014). Skin nerve alpha-synuclein deposits: a biomarker for idiopathic Parkinson Disease response. Neurology 83, 1582. 10.1212/WNL.0000000000000973 25332447

[B16] Donadio V. Incensi A. Piccinini C. Cortelli P. Giannoccaro M. P. Baruzzi A.. (2016). Skin nerve misfolded -synuclein in pure autonomic failure and Parkinson disease. Ann. Neurol. 79, 306–316. 10.1002/ana.24567 26606657

[B17] Feng S. Cokus S. J. Zhang X. Chen P.-Y. Bostick M. Goll M. G.. (2010). Conservation and divergence of methylation patterning in plants and animals. Proc. Natl. Acad. Sci. U.S.A. 107, 8689–8694. 10.1073/pnas.1002720107 20395551 PMC2889301

[B18] Grozdanov V. Bliederhaeuser C. Ruf W. P. Roth V. Fundel-Clemens K. Zondler L.. (2014). Inflammatory dysregulation of blood monocytes in Parkinson's disease patients. Acta Neuropathol. 128, 651–663. 10.1007/s00401-014-1345-4 25284487 PMC4201759

[B19] Gusella J. F. MacDonald M. E. Lee J.-M. (2014). Genetic modifiers of Huntington's Disease. Move. Disord. 29, 1359–1365. 10.1002/mds.26001 25154728

[B20] Hogenbirk M. A. Heideman M. R. Velds A. van den Berk P. C. M. Kerkhoven R. M. van Steensel B.. (2013). Differential programming of B cells in AID deficient mice. PLoS ONE 8:e69815. 10.1371/journal.pone.0069815 23922811 PMC3726761

[B21] Houlden H. Singleton A. B. (2012). The genetics and neuropathology of Parkinson's disease. Acta Neuropathol. 124, 325–338. 10.1007/s00401-012-1013-5 22806825 PMC3589971

[B22] Ide K. Yamada H. Umegaki K. Mizuno K. Kawakami N. Hagiwara Y.. (2015). Lymphocyte vitamin C levels as potential biomarker for progression of Parkinson's disease. Nutrition 31, 406–408. 10.1016/j.nut.2014.08.001 25592020

[B23] Jelinek J. Liang S. Lu Y. He R. Ramagli L. S. Shpall E. J.. (2012). Conserved dna methylation patterns in healthy blood cells and extensive changes in leukemia measured by a new quantitative technique. Epigenetics 7, 1368–1378. 10.4161/epi.22552 23075513 PMC3528692

[B24] Jones A. Lechner M. Fourkala E.-O. Kristeleit R. Widschwendter M. (2010). Emerging promise of epigenetics and DNA methylation for the diagnosis and management of women's cancers. Epigenomics 2, 9–38. 10.2217/epi.09.47 22122746

[B25] Kalakonda N. Fischle W. Boccuni P. Gurvich N. Hoya-Arias R. Zhao X.. (2008). Histone H4 lysine 20 monomethylation promotes transcriptional repression by L3MBTL1. Oncogene 27, 4293–4304. 10.1038/onc.2008.67 18408754 PMC2742506

[B26] Kaliszewski M. Knott A. B. Bossy-Wetzel E. (2015). Primary cilia and autophagic dysfunction in Huntington's disease. Cell Death Different. 22, 1413–1424. 10.1038/cdd.2015.80 26160070 PMC4532781

[B27] Krzywinski M. Schein J. Birol I. Connors J. Gascoyne R. Horsman D.. (2009). Circos: an information aesthetic for comparative genomics. Gen. Res. 19, 1639–1645. 10.1101/gr.092759.109 19541911 PMC2752132

[B28] Kumar A. Singh S. K. Kumar V. Kumar D. Agarwal S. Rana M. K. (2015). Huntington's disease: an update of therapeutic strategies. Gene 556, 91–97. 10.1016/j.gene.2014.11.022 25447911

[B29] Labadorf A. T. Myers R. H. (2015). Evidence of extensive alternative splicing in post mortem human brain HTT transcription by mRNA sequencing. PLoS ONE 10:e0141298. 10.1371/journal.pone.0141298 26496077 PMC4619731

[B30] Ladd-Acosta C. Fallin M. D. (2016). The role of epigenetics in genetic and environmental epidemiology. Epigenomics 8, 271–283. 10.2217/epi.15.102 26505319

[B31] Laird P. W. (2010). Principles and challenges of genome-wide dna methylation analysis. Nat. Rev. Genet. 11, 191–203. 10.1038/nrg2732 20125086

[B32] Legendre P. Legendre L. (2012). Numerical Ecology. 3rd Edn., Amsterdam: Elsevier Science BV.

[B33] Malik J. Getman M. Steiner L. A. (2015). Histone methyltransferase Setd8 represses Gata2 expression and regulates erythroid maturation. Mol. Cell. Biol. 35, 2059–2072. 10.1128/MCB.01413-14 25848090 PMC4438238

[B34] Marsh A. G. Pasqualone A. A. (2014). DNA methylation and temperature stress in an Antarctic polychaete, Spiophanes tcherniai. Front. Physiol. 5:173. 10.3389/fphys.2014.00173 24847277 PMC4017131

[B35] Masliah E. Dumaop W. Galasko D. Desplats P. (2013). Distinctive patterns of DNA methylation associated with Parkinson disease Identification of concordant epigenetic changes in brain and peripheral blood leukocytes. Epigenetics 8, 1030–1038. 10.4161/epi.25865 23907097 PMC3891683

[B36] McClay J. L. Shabalin A. A. Dozmorov M. G. Adkins D. E. Kumar G. Nerella S.. (2015). High density methylation qtl analysis in human blood via next-generation sequencing of the methylated genomic dna fraction. Gen. Biol. 16:291. 10.1186/s13059-015-0842-7 26699738 PMC4699364

[B37] Mehta S. H. Adler C. H. (2016). Advances in biomarker research in Parkinson's Disease. Curr. Neurol. Neurosci. Rep. 16:7. 10.1007/s11910-015-0607-4 26711276

[B38] Meissner A. (2015). (epi)genomics approaches and their applications. Methods 72, 1–2. 10.1016/j.ymeth.2014.12.011 25597875

[B39] Miller D. B. O'Callaghan J. P. (2015). Biomarkers of Parkinson's disease: Present and future. Metabol. Clin. Exp. 64, S40–S46. 10.1016/j.metabol.2014.10.030 25510818 PMC4721253

[B40] Nielsen C. H. Larsen A. Nielsen A. L. (2016). DNA methylation alterations in response to prenatal exposure of maternal cigarette smoking: A persistent epigenetic impact on health from maternal lifestyle? Arch. Toxicol. 90, 231–245. 10.1007/s00204-014-1426-0 25480659

[B41] Ordway J. M. Budiman M. A. Korshunova Y. Maloney R. K. Bedell J. A. Citek R. W.. (2007). Identification of novel high-frequency DNA methylation changes in breast cancer. PLoS ONE 2:e1314. 10.1371/journal.pone.0001314 18091988 PMC2117343

[B42] Pihlstrøm L. Berge V. Rengmark A. Toft M. (2015). Parkinson's disease correlates with promoter methylation in the alpha-synuclein Gene. Move. Disord. 30, 577–580. 10.1002/mds.26073 25545759

[B43] Poulopoulos M. Levy O. A. Alcalay R. N. (2012). The neuropathology of genetic Parkinson's disease. Move. Disord. 27, 831–842. 10.1002/mds.24962 22451330 PMC3383342

[B44] Pyle A. Anugrha H. Kurzawa-Akanbi M. Yarnall A. Burn D. Hudson G. (2016). Reduced mitochondrial DNA copy number is a biomarker of Parkinson's disease. Neurobiol. Aging 38, 216.e7–216.e10. 10.1016/j.neurobiolaging.2015.10.033 26639155 PMC4759605

[B45] Reyna-López G. Simpson J. Ruiz-Herrera J. (1997). Differences in dna methylation patterns are detectable during dimorphic transition of fungi by amplification of restriction polymorphisms. Mol. Genom. Genet. 253, 703–710. 10.1007/s004380050374 9079881

[B46] Robinson M. D. McCarthy D. J. Smyth G. K. (2010). edgeR: a Bioconductor package for differential expression analysis of digital gene expression data. Bioinformatics 26, 139–140. 10.1093/bioinformatics/btp616 19910308 PMC2796818

[B47] Rodríguez-Leyva I. Chi-Ahumada E. G. Carrizales J. Rodríguez-Violante M. Velazquez-Osuna S. Medina-Mier V.. (2016). Parkinson disease and progressive supranuclear palsy: protein expression in skin. Ann. Clin. Transl. Neurol. 3, 191–199. 10.1002/acn3.285 27042679 PMC4774258

[B48] Schneider S. A. Boettner M. Alexoudi A. Zorenkov D. Deuschl G. Wedel T. (2016). Can we use peripheral tissue biopsies to diagnose Parkinson's disease? a review of the literature. Eur. J. Neurol. 23, 247–261. 10.1111/ene.12753 26100920

[B49] Sudmant P. H. Rausch T. Gardner E. J. Handsaker R. E. Abyzov A. Huddleston J.. (2015). An integrated map of structural variation in 2,504 human genomes. Nature 526, 75–81. 10.1038/nature15394 26432246 PMC4617611

[B50] Tan Y. Wu L. Li D. Liu X. Ding J. Chen S. (2016). Methylation status of DJ-1 in leukocyte DNA of Parkinson's disease patients. Transl. Neurodegen. 5:5. 10.1186/s40035-016-0052-6 27034775 PMC4815061

[B51] Tan Y.-Y. Wu L. Zhao Z.-B. Wang Y. Xiao Q. Liu J.. (2014). Methylation of alpha-synuclein and leucine-rich repeat kinase 2 in leukocyte DNA of Parkinson's disease patients. Parkins. Relat. Disord. 20, 308–313. 10.1016/j.parkreldis.2013.12.002 24398085

[B52] van Esterik J. C. J. Vitins A. P. Hodemaekers H. M. Kamstra J. H. Legler J. Pennings J. L. A.. (2015). Liver DNA methylation analysis in adult female C57BL/6JxFVB mice following perinatal exposure to bisphenol A. Toxicol. Lett. 232, 293–300. 10.1016/j.toxlet.2014.10.021 25455458

[B53] Weitzel K. W. Alexander M. Bernhardt B. A. Calman N. Carey D. J. Cavallari L. H.. (2016). The IGNITE network: a model for genomic medicine implementation and research. BMC Med. Genom. 9:1. 10.1186/s12920-015-0162-5 26729011 PMC4700677

[B54] West L. E. Roy S. Lachmi-Weiner K. Hayashi R. Shi X. Appella E.. (2010). The MBT repeats of L3MBTL1 link SET8-mediated p53 methylation at lysine 382 to target gene repression. J. Biol. Chem. 285, 37725–37732. 10.1074/jbc.M110.139527 20870725 PMC2988377

[B55] Wickham H. (2009). **g**gplot2: Elegant Graphics for Data Analysis. New York, NY: Springer-Verlag.

[B56] Zhou X. Lindsay H. Robinson M. D. (2014). Robustly detecting differential expression in RNA sequencing data using observation weights. Nucl. Acids Res. 42:e91 10.1093/nar/gku310 24753412 PMC4066750

[B57] Zwagerman N. T. Richardson R. M. (2013). Neuropathology indicates a need for earlier neurorestorative intervention in Parkinson's Disease. Neurosurgery 73, N14–N15. 10.1227/01.neu.0000438333.49696.12 24257338

