# Supplementary figures and images for: Epigenetic DNA Methylation Profiling with MSRE: A Quantitative NGS Approach Using a Parkinson's Disease Test Case
Source: Front Genet. 2016 Nov 2;7:191. doi: 10.3389/fgene.2016.00191 (PMC5090125; doi:10.3389/fgene.2016.00191)

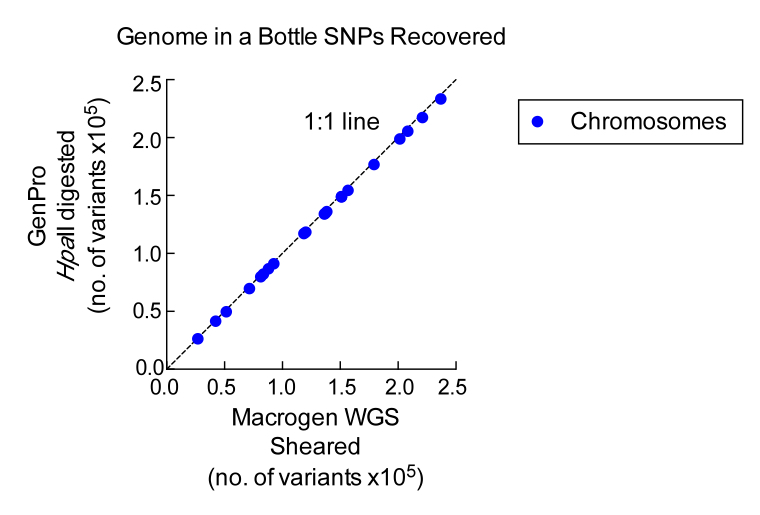

Supplement: Figure S1 — Efficiency of Genome Variant Analyses. The NIST standard “Genome in a Bottle” was processed using a commercial sequencing lab (Macrogen USA) comparing standard whole genome library preparation and downstream variant analysis (WGS) to a HpaII fragmentation prior to library preparation and then normal sequencing and variant analysis (GenPro). Both methods recovered 94% of the known SNP variants in the Genome in a Bottle genotype. [file Image1.JPEG]

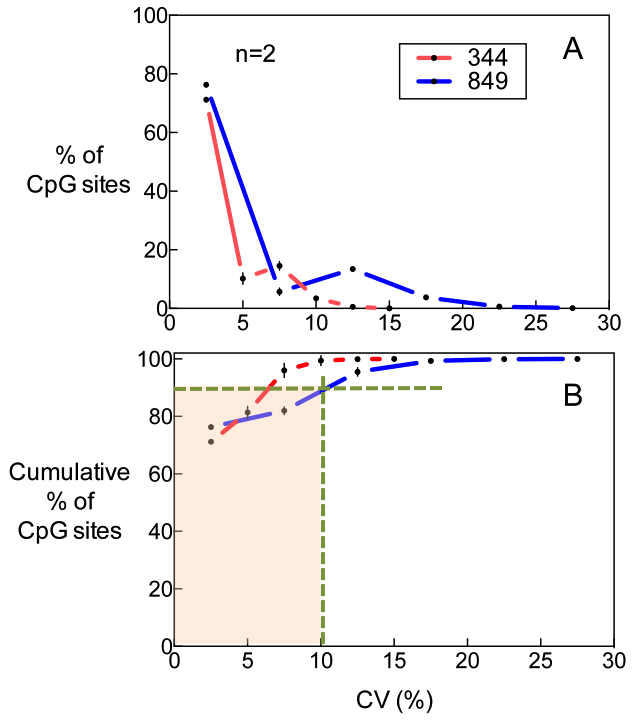

Supplement: Figure S2 — Replicate Precision. The gDNA from two separate samples were each split and used for replicate library preparations and NGS sequencing runs. (A) Frequency distribution of the observed Coefficient's of Variation (CV) for individual CpG sites. (B) Cumulative frequency plot showing that almost 90% of the measured CpG sites in the replicates had a CV < 10%. [file Image2.JPEG]
